# Preclinical evaluation of OMVs as potential vaccine candidates against *Salmonella enterica* serovar Enteritidis infection

**DOI:** 10.3389/fcimb.2022.1037607

**Published:** 2022-10-27

**Authors:** Xi Jiang, Chao Chu, Zhenyu Wang, Jiaojie Gu, Yaming Hong, Qiuchun Li, Xinan Jiao

**Affiliations:** ^1^ Key Laboratory of Prevention and Control of Biological Hazard Factors (Animal Origin) for Agri-Food Safety and Quality, Ministry of Agriculture of China, Yangzhou University, Yangzhou, China; ^2^ Jiangsu Key Lab of Zoonosis/Jiangsu Co-Innovation Center for Prevention and Control of Important Animal Infectious Diseases and Zoonoses, Yangzhou University, Yangzhou, China; ^3^ Joint International Research Laboratory of Agriculture and Agri-Product Safety, Yangzhou University, Yangzhou, China

**Keywords:** outer-membrane vesicles (OMVs), *Salmonella enterica* serovar Enteritidis (*S*. Enteritidis), *rfaQ*, *tolR*, vaccine

## Abstract

*Salmonella enterica* serovar Enteritidis is the most prevalent serotype that causes human infections worldwide. Consumption of *S.* Enteritidis-contaminated animal foods is a major source of human infections; however, eradicating bacteria from animals remains difficult. Therefore, it is necessary to develop new measures to prevent and control salmonellosis. Here, we used the outer-membrane vesicles (OMVs) of *S.* Enteritidis and assessed their protective efficacy and immune response in mice. Deletion of *tolR* in *S.* Enteritidis increased the production and size of OMVs compared to those in the wild type (WT) and Δ*rfaQ* strains. Intramuscular immunization with OMVs conferred greater protection than intraperitoneal and intranasal immunization. Moreover, OMVs extracted from both WT and Δ*tolR* strains provided an 83.3% protective rate in mice challenged with *S.* Enteritidis, which was higher than that provided by OMVs extracted from the Δ*rfaQ* strain. However, compared with OMVs from the Δ*tolR* strain, OMVs from WT and Δ*rfaQ* strains rapidly eradicated *S.* Enteritidis colonizing the liver, spleen, ileum, and cecum of BALB/c mice after immunization. Immunization with OMVs from each of the three strains induced humoral immune responses and showed no side effects on the growth of mice. Our study revealed that OMVs from various *S.* Enteritidis strains could be developed for use as subunit vaccine candidates against nontyphoidal *Salmonella* infections in mammals.

## Introduction

*Salmonella* is the major foodborne pathogen that causes human infections and has over 2600 serovars, including the serovars causing enteric fevers and the non-typhoidal *Salmonella* (NTS) serovars causing invasive disease and diarrheal disease (Mezal et al., 2014). The global burden of invasive non-typhoidal *Salmonella* (iNTS) diseases in 2019 was estimated to be 594,000 cases, 79,000 deaths and 6.11 million global disability-adjusted life years (DALYs) (Institute for Health Metrics and Evaluation (IHME), 2019). Globally, *Salmonella enterica* serovar Enteritidis (*S*. Enteritidis) has surpassed *S.* Typhimurium as the predominant serovar among all reported human NTS isolates globally in recent years (CDC (Centers for Disease Control and Prevention), 2018; EFSA and ECDC (European Food Safety Authority and European Centre for Disease Prevention and Control), 2020). The consumption of *S.* Enteritidis-contaminated animal foods is considered the major source of many human outbreaks. Poultry and poultry products have been reported to be the most common carriers of *S.* Enteritidis (Li et al., 2021; Wang et al., 2021). Controlling the spread of *S.* Enteritidis in poultry remains a major challenge. Therefore, it is crucial to develop new measures to prevent and control *S.* Enteritidis infection in humans.

Vaccines have been successfully used to protect against typhoid fever caused by *S.* Typhi; these include the capsular polysaccharide vaccine based on Vi antigen, and the live attenuated oral vaccine Ty21a (Germanier and Füer, 1975; Hessel et al., 1999). The success of these vaccines has promoted the development of NTS vaccine candidates, including live-attenuated, subunit-based, and recombinant protein-based vaccines (Hindle et al., 2002). The early-developed live-attenuated WT05 candidate showed good tolerability and induced strong antibody responses against LPS in some healthy volunteers; however, prolonged bacterial shedding in feces prevented the further application of this vaccine candidate (Hindle et al., 2002; Higginson et al., 2021). Glycoconjugate vaccines have also been developed against NTS strains, for example, the trivalent vaccine CVD1000 (trivalent COPS:FliC and TCV) in phase 1 (https://clinicaltrials.gov/ct2/show/NCT03981952). The CVD1000 vaccine consisted of core O-polySaccharides (COPS) from iNTS serovars (*S*. Typhimurium and *S.* Enteritidis), coupled with the carrier flagellin FliC and a Vi-tetanus toxoid (Baliban et al., 2018). Outer membrane vesicles (OMVs) or GMP-quality OMVs, considered as Generalized Modules for Membrane Antigens (GMMAs), are another promising vaccine candidates (Baliban et al., 2020). GMMAs are OMVs derived from genetically engineered bacteria to enhance the release of OMVs (Micoli et al., 2020). A GMMA-based vaccine against iNTS diseases will be tested in Phase 1 clinical trials in Europe and sub-Saharan Africa (https://cordis.europa.eu/project/id/815439). Immunization with *S.* Enteritidis and *S.* Typhimurium GMMA could induce Th1, Th2 and Th17 immune responses in mice and rabbits (Koeberling et al., 2014; Fiorino et al., 2021), which are crucial for the elimination of *Salmonella* cells *in vivo*. In OMVs from *S.* Enteritidis, 108 proteins were identified and 49.9% of proteins were enriched compared with bacterial cells (Liu et al., 2017). Immunization with OMVs in mice elicits antibodies against outer membrane proteins and LPS (Liu et al., 2017).

Gram-negative bacteria can spontaneously release OMVs in relatively low amounts and contain endotoxins (Nevermann et al., 2019). A previous study revealed that deletion of *tolR* caused high expression of OMVs in *S.* Typhi, but only 2-fold more OMVs in *S.* Typhimurium (Nevermann et al., 2019). The Tol-Pal system in gram-negative bacteria is a multiprotein composite that links the inner and outer membranes to maintain envelope integrity (Cascales et al., 2002). It comprises TolA, TolB, TolQ, TolR, and Pal protein. TolR is an inner membrane protein involved in the motility of *Salmonella* and *E. coli* (Cascales et al., 2002; Nevermann et al., 2019). RfaQ is a transferase that adds HepIII residue to LPS and is involved in the formation of a stable outer membrane (Li et al., 2018). Heptose residues in LPS are involved in LPS/TLR4 signaling by interacting with TLR4 residues (Cochet and Peri, 2017). Deletion of *rfaQ* in *S.* Enteritidis caused decreased bacterial virulence in both macrophages and animals (Yethon et al., 1998). Therefore, to enhance the production of OMVs and reduce inflammatory damage induced by OMVs, we selected *tolR* and *rfaQ* as target genes to construct *S.* Enteritidis mutants and evaluated the yield of OMVs produced by *S.* Enteritidis (WT, Δ*rfaQ* and Δ*tolR*) and identified the best immunization method appropriate for OMVs vaccination in mice. We determined the protective efficacy of OMVs from three *Salmonella* strains (WT, Δ*rfaQ* and Δ*tolR*) in BALB/c mice after challenge with *S.* Enteritidis. We evaluated the immune responses in OMVs-immunized mice and assessed the ability to eliminate bacteria after challenge with *S.* Enteritidis. The development of OMVs from *S.* Enteritidis strains for use as vaccine candidates has shown good application prospects in mammals.

## Materials and methods

### Mice

Six-to-eight weeks old BALB/c mice were purchased from Charles River Labs (Beijing, China) for animal experiments. All experiments were performed according to the guidelines of the Institutional Administrative Committee and Ethics Committee of Laboratory Animals, and were approved by the Animal Welfare and Ethics Committee of Yangzhou University (NSFC2019-SJXY-4). All efforts were made to minimize the suffering of mice.

### Construction of *S.* Enteritidis CMCC50041Δ*tolR* mutant

To promote the output of OMVs from *S.* Enteritidis, *tolR* was deleted from the *S.* Enteritidis CMCC50041 strain using the λ-RED mutation system, as described previously (Datsenko and Wanner, 2000). Briefly, the *tolR-cm^r^*-F/R primer pair was used to amplify the chloramphenicol resistance gene *cm^r^*, which was then transformed into the CMCC50041 strain carrying the pKD46 plasmid. The addition of L-arabinose (30 mM) induced recombinase expression in the pKD46 plasmid to promote homologous recombination in the CMCC50041 genome. Colonies grown on a Luria-Bertani (LB) agar plate (Thermo Fisher Scientific, USA) with 25μg/ml chloramphenicol and L-arabinose were CMCC50041Δ*tolR::cm^r^*. The subsequent transformation of the pCP20 plasmid into the bacterial strain expressing flippase resulted in the eradication of *cm^r^* to generate the mutant strain Δ*tolR*. All the primers used for the construction of CMCC50041Δ*tolR* in this study are shown in **Table S1**.

### Bacterial strains, media and growth conditions

The CMCC50041 strain was purchased from the China Institute of Veterinary Drug Control. The CMCC50041 *rfaQ* deleted strain Δ*rfaQ* was constructed and preserved in our laboratory (Li et al., 2018). All *Salmonella* strains preserved in the -80°C freezer were recovered on LB agar plates at 37°C for overnight cultivation. All the strains used in this study are listed in **Table S1**.

### Extraction of OMVs from *S*. Enteritidis

A single colony was obtained from the LB agar plate and inoculated into fresh LB medium at 37°C for overnight cultivation. The extraction of OMVs from different *S.* Enteritidis strains was performed using the Bacterial MVs Isolation Kit (Rengen Biosciences, Liaoning, China) according to the manufacturer’s instructions. Briefly, when the OD_600_ reached 1.5, 40 ml of bacterial culture medium was collected and subjected to centrifugation at 5,000×g for 20 min at 4°C. The supernatant was transferred to a fresh tube for centrifugation under the same conditions to remove cellular debris completely. The supernatant was then mixed with 4 ml Binding Buffer and 1.6 ml Binding Resin for 15 min, followed by centrifugation at 1,500×g for 2 min. After removing the supernatant, 1 ml of the pellet was transferred to a spin column for 2 min, followed by centrifugation at 2,000×g for 2 min. After washing twice with 2 ml of Washing Buffer, the column was incubated with 1.5 ml of OMV Elution Buffer (HEPES buffer) for 2min and centrifuged at 3,000×g for 2 min twice. OMVs extraction was performed in triplicate for each strain. The eluted OMVs were subjected to concentration analysis and preserved in a -80°C freezer.

### Characterization of OMVs

The protein concentration in the extracted OMVs was measured using a BCA Protein Assay kit (Beyotime, Jiangsu, China). OMV proteins, including outer membrane proteins (Omps), were visualized using SDS-PAGE of 10 μl extracted OMVs. The total lipid concentration of the extracted OMVs was determined using the fluorescent dye FM4-64. Fluorescence was measured at 515 nm (excitation) and 640 nm (emission) to obtain the relative fold of OMV yield in comparison with that of the WT strain. The OMV samples were placed onto a 200-mesh copper grid, negatively stained with 4% uranyl acetate and visualized using an HT7800 transmission electron microscope (TEM) to determine the size of the OMVs (Hitachi, Japan).

### OMVs immunization protocol and immune protection assessment

To obtain an efficient immunization route for OMVs, three immunization methods were used to inoculate OMVs from the WT strain: intramuscular (i.m.) immunization, intraperitoneal (i.p.) immunization and intranasal (i.n.) immunization. Six-to-eight-week-old BALB/c mice (n = 24) were divided into four groups (six mice per group). Each group was immunized with 20 μg (i.m. and i.p.) or 5 μg (i.n.) OMVs (100 μl) per mouse. The 20 μg or 5 μg concentration reflected the protein content of OMVs. The second immunization was performed with the same amount of OMVs two weeks after the first immunization. The group i.m. immunized with HEPES buffer was used as the negative control. Two weeks later, the mice were orally challenged with 2 × 10^7^ colony-forming unit (CFU) of *S.* Enteritidis CMCC50041. The number of surviving mice was recorded daily for two weeks to assess the relationship between protective efficacy and immunization methods. After identifying the best immunization method from the three aforementioned methods, i.m. was used to compare the protective efficacy of OMVs from the three strains (WT, Δ*rfaQ* and Δ*tolR*) in mice, as mentioned above.

### Immune response and growth of mice after immunization with OMVs

A total of 24 six-to-eight weeks old BALB/c mice were divided into four groups. Each group (n=6) was i.m. immunized twice with 20 μg of OMVs per mouse. After the first immunization, the body weights of the immunized mice were measured at 1, 3, 5, 7, 9, 11, and 13 days post-immunization (dpi) to compare the effects of OMVs from the three strains (WT, Δ*rfaQ*, and Δ*tolR*) on the growth of mice. To determine the immune response induced by immunization with OMVs, serum samples were collected from mice at 6 h after immunization. The cytokine levels in these samples were detected using the BD™ Cytometric Bead Array (CBA) Mouse Inflammation Kit (BD, USA). The humoral immune response was further assessed by measuring IgG titers specifically against the heat-killed WT strain using an enzyme-linked immunosorbent assay (ELISA). The CMCC50041 was used as the coating antigen (10^7^CFU/well). The WT strain was heat-killed and then added to a 96-well plate pretreated with 5% glutaraldehyde for overnight cultivation at 56°C. Serum samples were collected at 7, 14, 21, and 28 dpi and then serially diluted for 2 h incubation to serve as the primary antibody. The horseradish peroxidase (HRP)-conjugated goat anti-mouse IgG (Sigma-Aldrich, USA) at a 1:10,000 dilution was used as the secondary antibody for one hour of incubation. The activity of HRP was measured using 3,3^’^5,5’-tetramethylbenzidine (TMB, Solarbio, China) as the substrate, and the OD450 value was determined using a Sunrise™ absorbance microplate reader (Tecan, Swiss).

### Persistence and clearance of *Salmonella* in mice

To determine the persistence and eradication of *Salmonella* in immunized mice, 60 BALB/c mice aged 6-8 weeks were divided into four groups (n = 15) and immunized with OMVs from the three strains (WT, Δ*rfaQ* and Δ*tolR*), as mentioned above. Two weeks after the second immunization, the mice were orally challenged with 2 × 10^5^ CFU of *S.* Enteritidis CMCC50041. The HEPES buffer-immunized group challenged with the WT strain was used as the control group. Samples of the liver, spleen, Peyer’s patches, ileum, and cecum were obtained from five mice in each group at 7, 14 and 21 days post-challenge (dpc). The samples were suspended in PBS for homogenization, serially diluted and subsequently inoculated on a Brilliant Green Agar plate to count the number of bacteria in these tissues. The bacterial count is expressed as log10 CFU/g.

### Statistical analysis

All animal experiments were performed at least twice. Each graph represents the results of independent samples. All data are presented as mean ± standard error of the mean (SEM). The data were analyzed using GraphPad Prism software (version 8.0). The statistically significant differences between groups were determined using two-way ANOVA with Tukey’s test. Statistical significance was set than 0.05.

## Results

### Effect of *tolR* deletion on OMVs yield in *S*. Enteritidis

**Figure 1:**
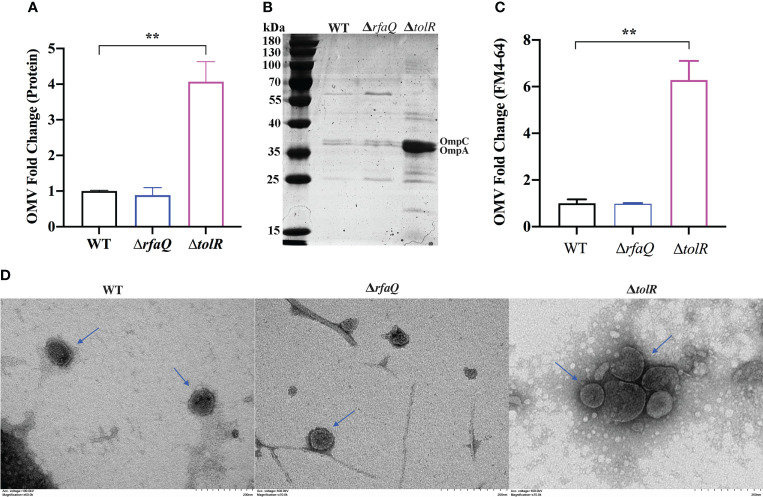
Comparison of OMV production by the three *S.* Enteritidis strains. **(A)** The relative fold of OMV production by the *S.* Enteritidis Δ*rfaQ* and Δ*tolR* strains was calculated and compared with that of the WT strain (normalized to 1) by measuring OMV protein concentrations. **(B)** SDS-PAGE of 10 μl OMVs extracted from WT, Δ*rfaQ*, and Δ*tolR* strains. **(C)** Measurement of OMV production by the WT, Δ*rfaQ*, and Δ*tolR* strains using the fluorescent dye FM4-64 to determine the OMV lipid concentrations. The lipid concentrations in OMVs from the WT strain were normalized to 1. **(D)** Determination of the size of OMVs from WT, Δ*rfaQ* and Δ*tolR* strains using TEM analysis. Scale bar = 200 nm. The protein and LPS measurements of OMVs were performed in triplicate and are shown as mean ± SEM. **p ≤ 0.001.

To extract OMVs from the *S.* Enteritidis WT, Δ*rfaQ* and Δ*tolR* strains, 40 ml overnight cultures of bacteria were prepared according to the manufacturer’s instructions. The average protein concentration in OMVs from the WT strain was approximately 0.206 mg/ml, which was similar to that in OMVs from the Δ*rfaQ* strain (0.182 mg/ml) (*p* > 0.05). However, the protein concentration in OMVs from the Δ*tolR* strain was 0.836 mg/ml, which was four-fold higher than that in OMVs from the WT strain (*p* ≤ 0.01) (**Figure 1A**). SDS-PAGE further revealed that two bands representing the outer membrane proteins OmpC and OmpA were evident in OMVs from the Δ*tolR* strain (**Figure 1B**). FM4-64 quantification of lipids revealed over six-fold enrichment in OMVs from the Δ*tolR* strain compared with those from the WT and Δ*rfaQ* strains (*p* ≤ 0.01) (**Figure 1C**). Moreover, TEM analysis revealed that the diameter of OMVs from WT and Δ*rfaQ* strains was 72.99 ± 30.74 nm and 60.78 ± 25.27 nm, respectively. OMVs from the Δ*tolR* strain had a diameter of 85.31 ± 32.93 nm and contained electron-dense structures with various shapes and sizes (**Figure 1D**). These results indicate that deletion of *tolR* enhances OMV production in *S*. Enteritidis.

### Identification of i.m. as the appropriate immunization method

To identify the appropriate immunization method for the administration of OMVs in mice, 20 μg OMVs from the WT strain were inoculated in each BALB/c mouse (n = 6) through the i.m. and i.p. methods, and 5 μg of the OMVs were inoculated through the i.n. method. After challenge with 10 LD_50_ (2 × 10^7^ CFU) of the CMCC50041 strain, 83.3% (n = 5/6) of the mice survived in the i.m. group, while only 50% survived in the i.p. group. No mice in the i.n. group survived (**Figure S1**). Hence, intramuscular injection was selected as the preferred immunization method in the subsequent analysis for immunization with 20 μg OMVs.

### Safety of OMVs as vaccine candidates

**Figure 2:**
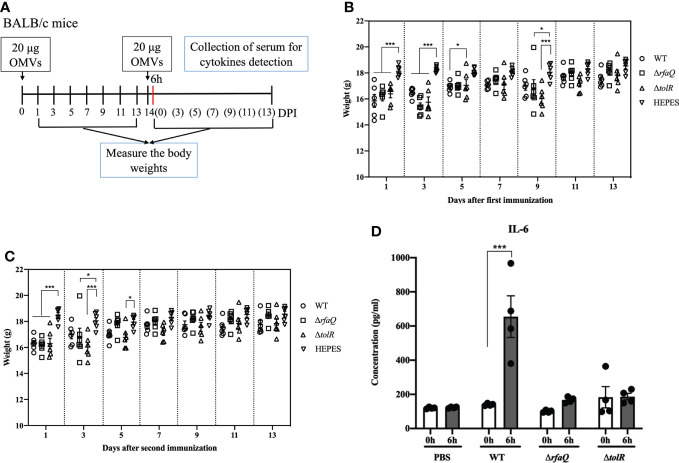
Assessment of the safety of OMVs on mouse growth. **(A)** Design of the animal experiment to assess the safety of OMVs **(B)** Body weights of mice after the first immunization with OMVs. **(C)** Growth of mice after the second immunization with OMVs. **(D)** IL-6 levels in mouse sera at 6 h after immunization with OMVs from the WT, Δ*rfaQ* and Δ*tolR* strains. Data are expressed as mean ± SEM. *p ≤ 0.05; ***p ≤ 0.001.

After the first immunization with OMVs from the three strains, the body weights of the mice were significantly decreased at 1 and 3 dpi compared with those of the mice in the HEPES buffer-immunized group (*p* ≤ 0.001) (**Figures 2A, B**). However, the mice recovered gradually and no significant difference was noted among all groups at 11 and 13 dpi (*p* > 0.05) (**Figure 2B**). At 5 dpi, the group immunized with OMVs from the Δ*tolR* strain showed significantly increased body weight compared with the group immunized with OMVs from the WT strain (*p* ≤ 0.05) (**Figure 2B**). After the second immunization, the body weight of the mice in the three groups significantly decreased at 1 dpi compared to that of the mice in the HEPES buffer-immunized group (*p* ≤ 0.001). However, the mice recovered gradually and no significant difference was noted among the groups at 7 dpi (*p* > 0.05) (**Figure 2C**). These results indicated that immunization with OMVs caused acute reactions in mice but recovered quickly. At 6h after the second immunization, the level of IL-6 dramatically increased in the sera of mice immunized with OMVs from the WT strain (*p* ≤ 0.001); however, it did not increase in the sera of mice immunized with OMVs from the Δ*rfaQ* and Δ*tolR* strains (*p* > 0.05) (**Figure 2D**). No significant difference was noted in the levels of TNF-α among the three OMV-immunized groups (*p* > 0.05) (**Figure S2**).

### Antibody responses induced by OMVs

**Figure 3:**
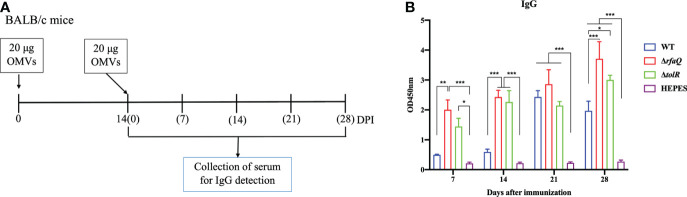
Antibody responses induced by *S.* Enteritidis OMVs. **(A)** Design of the animal experiment to detect serum IgG levels induced by OMVs. **(B)** IgG antibody against whole bacterial lysates was detected in serum samples from OMV-immunized mice at 7, 14, 21, and 28 days after immunization. The serum was diluted to 1:800 to assess IgG levels using the lysate of the CMCC50041 strain as a coating antigen in ELISA. The IgG levels in the serum samples were measured at 450 nm. Data are expressed as mean ± SEM. *p ≤ 0.05; **p ≤ 0.01; ***p ≤ 0.001.

To identify the immune response induced by immunization with different OMVs, we detected the antibodies (IgG) against *S.* Enteritidis and assessed the cytokines in the serum samples from the immunized mice (**Figure 3A**). The sera from the mice were diluted 1:800 and analyzed at OD450 nm. As shown in the **Figure 3B**, compared with the HEPES buffer-immunized mice, the levels of IgG against *Salmonella* significantly increased in the mice immunized with OMVs from the Δ*rfaQ* (*p* ≤ 0.001) and Δ*tolR* strains (*p* ≤ 0.05 at 7 dpi; *p* ≤ 0.001 at 14 dpi). However, no significant difference was observed in mice immunized with OMVs from the WT strain after the first immunization (*p* > 0.05). After the second immunization, the antibodies against *S.* Enteritidis dramatically increased in all OMV-immunized groups (*p* ≤ 0.001), indicating that immunization with OMVs could induce strong humoral immunity against *S.* Enteritidis.

### Protective efficacy of the OMVs-immunized mice against *S*. Enteritidis infection

**Figure 4:**
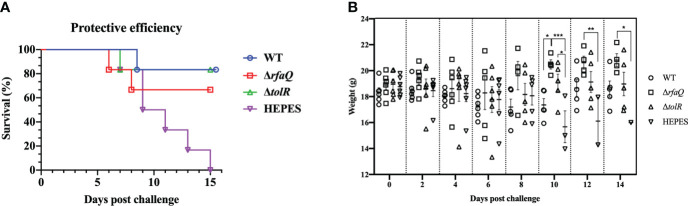
Protective efficacy of OMVs produced by three *S.* Enteritidis strains in mice. Each of the six BALB/c mice in the three groups was i.m. immunized with OMVs from the WT, Δ*rfaQ*, and Δ*tolR* strains. Another control group were immunized with HEPES buffer. After challenge with CMCC50041, the number of surviving mice was monitored for 15 days. Protective efficacy was determined by the survival rate of mice in each group after challenge with the CMCC50041 strain **(A)**. Body weights of mice after challenge with the CMCC50041 strain at 2, 4, 6, 8, 10, 12, and 14 dpi **(B)**. Data are expressed as mean ± SEM. *p ≤ 0.05; **p ≤ 0.01; ***p ≤ 0.001.

As intramuscular injection was identified as the best immunization method, we determined the protective efficacy of OMVs from the WT, Δ*rfaQ* and Δ*tolR* strains in mice. As shown in **Figure 4A**, no mouse in the HEPES-buffer-immunized group survived the challenge with the WT strain. In the group immunized with OMVs from the Δ*rfaQ* strain, one mouse each died at 6 and 8 dpc, with a protective efficacy of 66.7%. In the groups immunized with OMVs from the WT and Δ*tolR* strains, the protective efficacy was 83.3%, which was higher than that in the group immunized with OMVs from the Δ*rfaQ* strain. Moreover, five out of six mice survived in the two groups immunized with OMVs from the WT or Δ*tolR* strain, indicating that OMVs from the WT strain could provide strong protection against *S.* Enteritidis infection. In addition, we measured the body weight of the mice in each group after the challenge. There was no change in the body weight of the mice at 2, 4, 6, and 8 dpc (*p* > 0.05). Due to the death of the mice or severe infections, the body weight of the mice dramatically decreased in the HEPES buffer-immunized group compared to that of the mice in the group immunized with OMVs from the Δ*rfaQ* strain at 10 (*p* ≤ 0.001), 12 (*p* ≤ 0.01) and 14 (*p* ≤ 0.05) dpc (**Figure 4B**).

### Eradication of *S.* Enteritidis colonized in the OMVs-immunized mice

**Figure 5:**
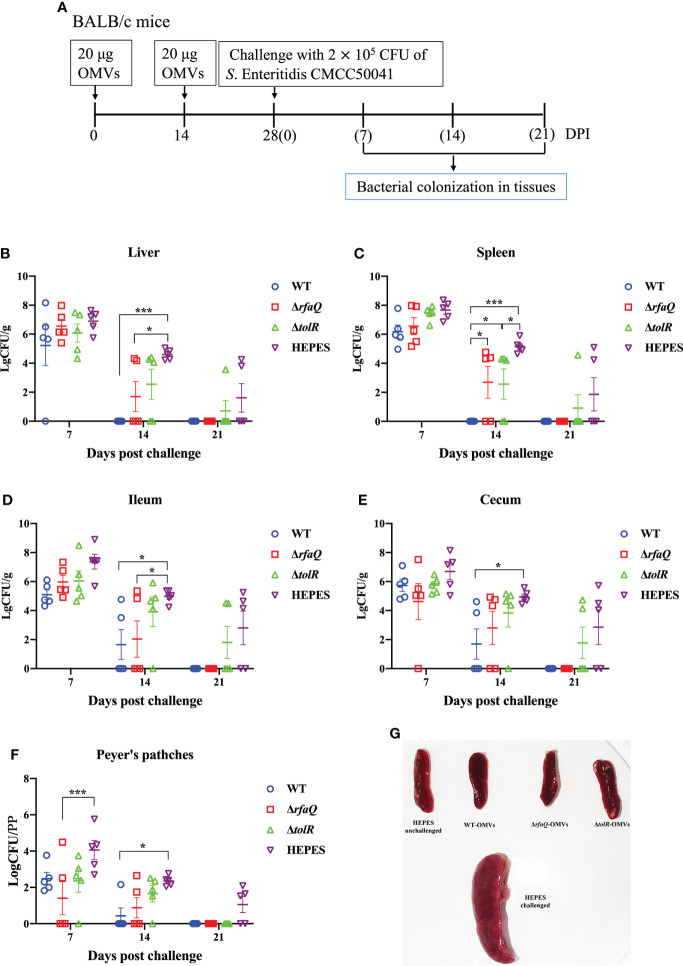
Eradication of *S.* Enteritidis colonization in mice. BALB/c mice were immunized with OMVs and challenged with the CMCC50041 strain **(A)**. Bacterial colonization in the liver **(B)**, spleen **(C)**, ileum **(D)**, cecum **(E)** and Peyer’s patches **(F)** was assessed using log CFU/g or log CFU/PP (for Peyer’s patches). Spleen swelling was assessed for comparison among the groups **(G)**. Data are expressed as the mean ± SEM. *p ≤ 0.05; ***p ≤ 0.001.

Persistent infection is a major characteristic of *S.* Enteritidis in humans and animals; therefore, the ability of the vaccine-immunized group to eradicate colonized *Salmonella* should be used to assess the effect of vaccines (**Figure 5A**). Compared to the HEPES buffer-immunized group, no significant decrease in bacterial colonization was noted in the three OMV-immunized groups at 7 dpc (*p* > 0.05). At 14 dpc, the bacteria were eradicated from the liver and spleen of mice immunized with OMVs from the WT strain, and bacterial colonization was significantly decreased in the ileum, cecum and Peyer’s patches of these mice (*p* ≤ 0.05) (**Figures 5A–F**). In the group immunized with OMVs from the Δ*rfaQ* strain, two or three mice showed decreased bacterial colonization in the liver, spleen, ileum, cecum and Peyer’s patches, whereas in the group immunized with OMVs from the Δ*tolR* strain, three or four mice showed a high bacterial load in the five tissues at 14 dpc. At 21 dpc, the bacteria were eradicated from the five tissues in the groups immunized with OMVs from the WT and Δ*rfaQ* strains. However, in the group immunized with OMVs from the Δ*tolR* strain, one or two mice showed bacterial colonization in the liver, spleen, ileum and cecum at 21 dpc (**Figures 5B–E**). The spleen samples from the HEPES-buffer-immunized group showed evident swelling after challenge with the WT strain, whereas the spleen samples from the OMVs-immunized groups were similar to those from the unchallenged group (**Figure 5G**). However, the group immunized with OMVs from the Δ*tolR* strain could not eradicate colonized bacteria from the ileum and cecum in two mice at 21 dpc (**Figures 5D, E**).

## Discussion

According to a report by the European Union, *S.* Enteritidis was the top serovar causing human salmonellosis from 2017 to 2020, accounting for approximately 50% of *Salmonella* infections in humans (EFSA and ECDC (European Food Safety Authority and European Centre for Disease Prevention and Control), 2020). *S.* Enteritidis was also the top serovar in the USA, accounting for 16.8% of clinically confirmed *Salmonella* infections in humans in 2016 (CDC (Centers for Disease Control and Prevention), 2018). Surveillance of human salmonellosis in China has revealed that *S.* Enteritidis is the predominant serovar causing iNTS infections (Zhan et al., 2019; Bao et al., 2020; Sun et al., 2021). To control iNTS infections caused by *S.* Enteritidis, it is necessary to develop an appropriate vaccine for humans. To date, no vaccine has been administered to humans to prevent iNTS infection. However, some vaccine candidates based on OMVs have displayed application prospects and will soon enter Phase I clinical trials (Koeberling et al., 2014; Fiorino et al., 2021).

OMVs are naturally secreted by most Gram-negative bacteria and can induce strong humoral and cellular immune responses (Liu et al., 2018). An OMV-based vaccine has been licensed for use against *Neisseria meningitidis* infections in humans (Sanders and Feavers, 2011). LPS is the main component of OMVs (Wang et al., 2020), which affects the safety of OMVs as vaccines. Therefore, the development of OMVs with reduced LPS content is a strategy to improve the safety of OMVs from *Salmonella*. For example, knockout of *msbB* in *S.* Typhimurium yielded low-endotoxic OMVs (Lee et al., 2009). In the present study, we used OMVs from the Δ*rfaQ* strain with a deficiency of HepIII residues on LPS without affecting the main structure of LPS (Yethon et al., 1998; Li et al., 2018). The deletion of *rfaQ* had no effect on the yield of OMVs, but decreased the inflammatory response and increased the protection of mice against *S.* Enteritidis infection, indicating that the improved OMVs from the Δ*rfaQ* strain could be developed as a vaccine for use in mammals. *tolR* belongs to the Tol-Pal system and is related to the integrity of the *Salmonella* cell wall membranes (Meloni et al., 2015; Rossi et al., 2016). Deletion of *tolR* results in the release of OMVs in large quantities and improves their yield, especially for OMVs cargo selection (Rossi et al., 2016; Micoli et al., 2018; Li et al., 2022). Our study confirmed that the Δ*tolR* strain produced more and larger OMVs than the WT and Δ*rfaQ* strains. Moreover, the protective efficacy of OMVs from the Δ*tolR* strain in mice was identical to that of OMVs from the WT strain indicating that deletion of *tolR* increased the yield of OMVs and preserved their protective ability against *S.* Enteritidis infection. However, OMVs from the Δ*tolR* strain displayed decreased ability to eliminate challenged bacteria in BALB/c mice, implying that these OMVs need to be improved in further research.

The improved protective efficacy of GMMA was closely related to the immune response induced by OMVs. The *S.* Typhimurium GMMA/Alhydrogel candidate vaccine can induce a high level of O:4,5-specific serum IgG that is maintained for more than 28 weeks and can produce long-lived plasma cells in the spleen and bone marrow (Fiorino et al., 2021). In the present study, *S.* Enteritidis OMVs induced high levels of serum IgG against the whole bacterial lysates for at least four weeks, indicating that OMVs could induce a strong and persistent humoral immune response (Micoli et al., 2018; Fiorino et al., 2021). Moreover, after challenge with WT *S.* Enteritidis, the OMV-immunized groups displayed a stable increase in body weight compared with the HEPES buffer-immunized group, with the group immunized with OMVs from the Δ*rfaQ* strain showing better results than the other two immunized groups. In addition to humoral immune responses, GMMA can induce strong cellular immune responses in mammals (Koeberling et al., 2014; Fiorino et al., 2021). The i.n. method induced a Th1-related immune response, subcutaneous injection induced a balanced Th1/Th2 profile and mixed immunization stimulated multifunctional Th1/Th17 CD4^+^ T cells (Koeberling et al., 2014; Baliban et al., 2020). Our study revealed that i.n. immunization with OMVs is not better than i.m. immunization, potentially because the i.n. immunization dose of OMVs (5μg) is lower than that (20μg) of the i.m. immunization. In addition, the i.m. immunization did not induce inflammatory responses in mice, indicating that OMVs from *S.* Enteritidis are safe for mice.

The overall eradication of *S.* Enteritidis in host tissues can prevent horizontal and vertical transmission of bacteria, which is a crucial index for evaluating the protective efficiency of vaccines (Tang et al., 2019). Live attenuated *Salmonella* vaccines have been confirmed to show a strong ability to eliminate pathogens in the liver, spleen, and ileum but not in the cecum (Li et al., 2019). Reported subunit vaccines such as FliC or SPI-1 effectors also had limitations in clearing *Salmonella* in the cecum (Desin et al., 2011; Wisner et al., 2011; Okamura et al., 2012). This study revealed that OMVs from *S.* Enteritidis WT and Δ*rfaQ* strains could clear bacterial colonization in the cecum. OMVs also exhibit potent adjuvant effects (Liu et al., 2018). Therefore, OMVs can be used directly to immunize hosts without mixing with other adjuvants.

## Conclusions

In summary, we evaluated the characteristics of OMVs from WT, Δ*rfaQ*, and Δ*tolR S.* Enteritidis strains and compared their protective efficacy against *S.* Enteritidis infection in BALB/c mice. Deletion of *tolR* increased the yield of OMVs, conferring a high level of protection against *Salmonella* infection. Compared with OMVs from the Δ*rfaQ* and Δ*tolR* strains, OMVs from the WT strain resulted in rapid clearance of challenged *S.* Enteritidis from mouse tissues after immunization. However, the group immunized with OMVs from the Δ*rfaQ* strain showed a stable increase in body weight and a decreased inflammatory response in mice compared to OMVs from the WT strain. These OMVs from *S.* Enteritidis can be developed as vaccine candidates against iNTS infections in mammals.

## Data availability statement

The original contributions presented in the study are included in the article/**Supplementary Material**. Further inquiries can be directed to the corresponding authors.

## Ethics statement

The animal study was reviewed and approved by Animal Welfare and Ethics Committee of Yangzhou University.

## Author contributions

QL and XaJ contributed to conception and design of the study. XJ and CC performed the experiments and collected the data. ZW, JG, and YH performed the analysis. XJ and CC wrote the first draft of the manuscript. QL and XaJ revised the manuscript. All authors contributed to manuscript revision, read, and approved the submitted version.

## Funding

This research was supported by National Natural Science Foundation of China (31920103015, 31730094, 32072821), The fifth phase of “333 project” scientific research project in Jiangsu Province (BRA2020002), and The Priority Academic Program Development of Jiangsu Higher Education Institution (PAPD).

## Conflict of interest

The authors declare that the research was conducted in the absence of any commercial or financial relationships that could be construed as a potential conflict of interest.

## Publisher’s note

All claims expressed in this article are solely those of the authors and do not necessarily represent those of their affiliated organizations, or those of the publisher, the editors and the reviewers. Any product that may be evaluated in this article, or claim that may be made by its manufacturer, is not guaranteed or endorsed by the publisher.
