# Supplementary material for: Preclinical evaluation of OMVs as potential vaccine candidates against Salmonella enterica serovar Enteritidis infection
Source: Front Cell Infect Microbiol. 2022 Oct 27;12:1037607. doi: 10.3389/fcimb.2022.1037607 (PMC9646977; doi:10.3389/fcimb.2022.1037607)

Supplementary Material

**Supplementary Table 1** Bacterial strains, plasmids and primers used in this study

| **Strains, plasmids and primers** | **Description of Genotype** | **Source (reference)** |
| --- | --- | --- |
| *S.* Enteritidis |  |  |
| CMCC50041 | Wild-type strain | China Institute of Veterinary Drug Control |
| Δ*rfaQ* | *rfaQ* deleted in CMCC50041 | (Li et al., 2018) |
| Δ*tolR* | *tolR* deleted in CMCC50041 | This work |
| *E. coli* |  |  |
| χ7213 | Donor strain, *Km^R^* | Our laboratory |
| Plasmids |  |  |
| pKD3 | chloramphenicol resistance cassette | (Datsenko and Wanner 2020) |
| pKD46 | *Ap^R^*, λ-Red mutation system | (Datsenko and Wanner 2020) |
| pCP20 | Temperature-sensitive Flp recombinase plasmid | (Datsenko and Wanner 2020) |
| Primers |  |  |
| *tolR-cm^r^*-F | CGCGATTCTGCACCGCCAGGCGTTTACCGTAAGCGAAAGCAACAAGGGGTAAGCCATGTGTGTAGGCTGGAGCTGCTTCG | 429 bp |
| *tolR-cm^r^*-R | CCAAAAAACTGTTCGCCTGTTACTCGCCGTCTTTCAAGCCAACGGGACGCAGACTTCACATATGAATATCCTCCTTAG |  |
| *tolR-*Out-F | CTTTATTTATCTTCCAGAGGGAC | 864 bp in wild type strain  436 bp in mutant strain |
| *tolR-*Out-R | CGATAATCTTATAGCCCGTA |  |
| *tolR*-In-F | ATGTCAACAGGATTACGTTTC | 221 bp |
| *tolR-*In-R | TCAGTACATGATCAGATCCGATGG |  |

**Figure S1.** Evaluation of three immunization methods Intramuscular (i.m.) immunization, intraperitoneal (i.p.) immunization and intranasal (i.n.) immunization methods were used to assess the efficiency of OMVs as vaccine candidates in six BALB/c mice per group. A group of six mice immunized with HEPES buffer was used as the negative control. The number of surviving mice was counted after challenge with 2 × 10^7^ CFU of the CMCC50041 strain.


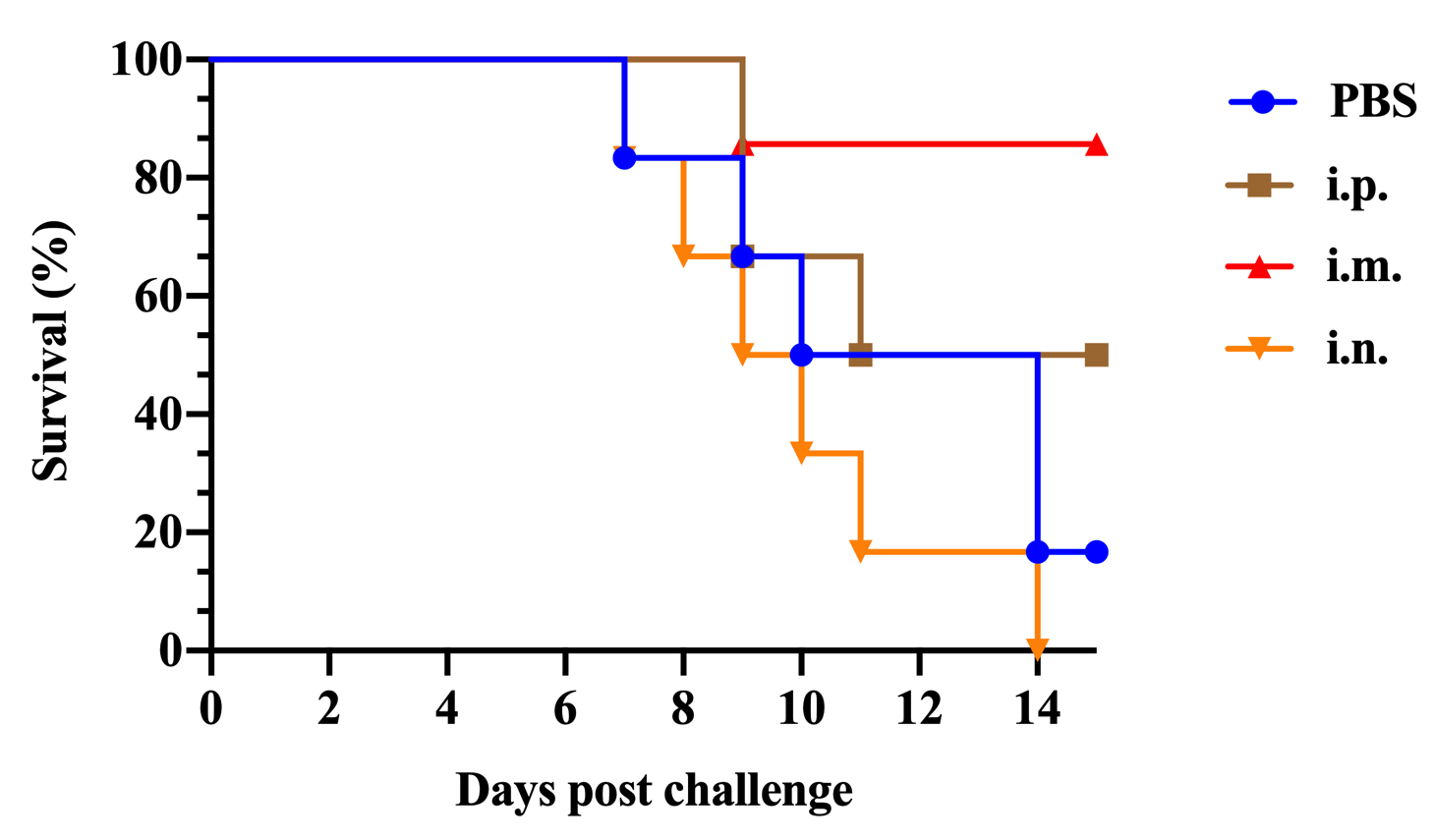


**Figure S2.** The TNF-α level in mouse sera at 6 h after immunization with OMVs from WT, Δ*rfaQ* and Δ*tolR* strains.


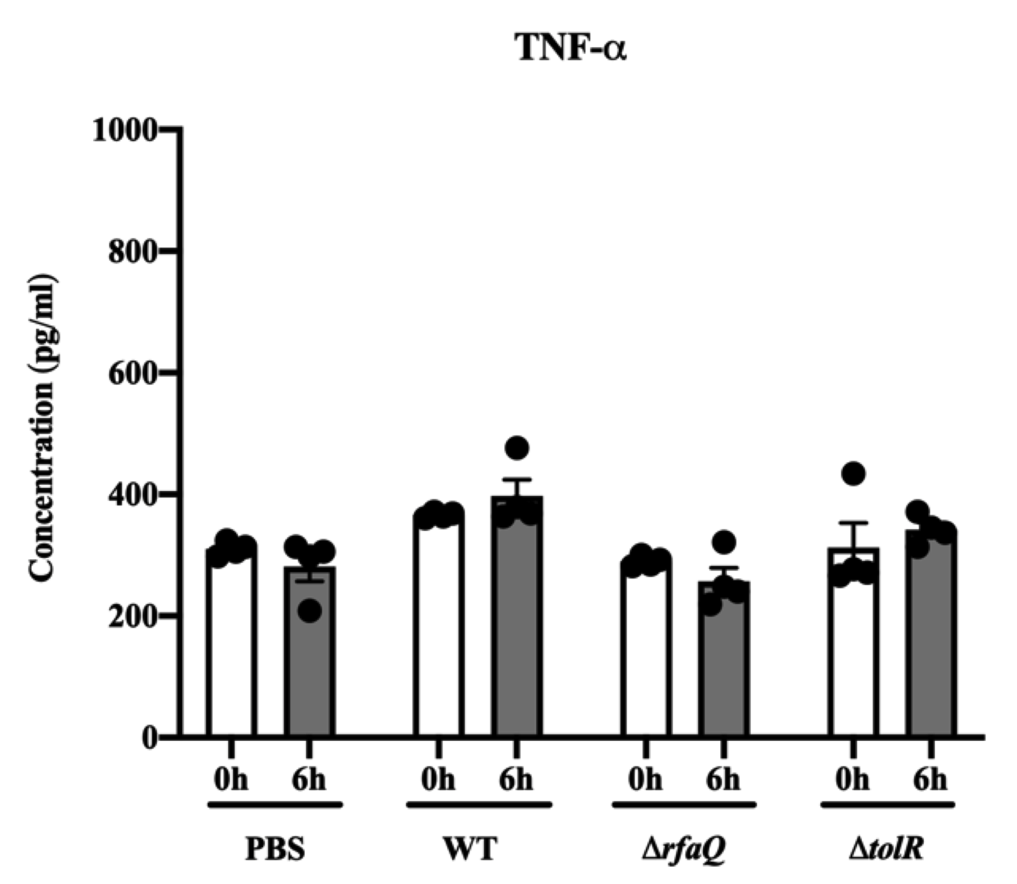

Supplement: Supplementary file 1 [file DataSheet_1.docx]
